# Prehospital times and outcomes for hypotensive trauma patients in Sweden

**DOI:** 10.1186/s13049-026-01597-2

**Published:** 2026-03-24

**Authors:** Axel Limbäck, Oscar Lapidus, Denise Bäckström

**Affiliations:** 1 Department of Biomedical and Clinical Sciences, Linköping University, Linköping, Sweden; 2 Department of Clinical Science, Intervention and Technology, Karolinska Institutet, Stockholm, Sweden; 3 Department of Leadership and Command & Control, Swedish Defence University, Karlstad, Sweden

## Abstract

**Background:**

The influence of EMS transport time on trauma patient survival has previously been examined. Studies have shown conflicting results, and some studies have been unable to prove any association between prehospital times and outcomes for trauma patients. In the context of Swedish trauma systems, the impact of prehospital time remains unclear.

**Aim:**

The aim of the present study was to investigate the correlation between prehospital time and outcomes for hypotensive trauma patients in Sweden.

**Methods:**

A total of 3,740 hypotensive trauma patients treated during the years 2013–2022 were identified through the Swedish Trauma Registry (SweTrau). The study included both ground ambulance patients and helicopter emergency medical services patients. A binary logistic regression model was used to analyse the correlation between total prehospital time and mortality while controlling for confounding factors. The primary study endpoint of this study was 30-day mortality after the initial traumatic event.

**Results:**

After adjusting for potentially confounding factors, including RTS, NISS, patient age, sex, and methods of injury, a longer total prehospital time was found to be associated with increased odds of mortality in the study population. In the present study, a one-minute increase in total prehospital time was found to have an adjusted odds ratio (AOR) of 1.005 for mortality (95% CI 1.000–1.009; *P* = 0.046) for undifferentiated hypotensive trauma patients.

**Conclusion:**

In this nationwide study aimed at investigating the correlation between prehospital time and survival among hypotensive trauma patients in Sweden, a longer total prehospital time was found to be associated with increased odds of mortality for undifferentiated hypotensive trauma patients, with the odds of mortality increasing by 5.1% per 10-min increase in total prehospital time.

## Background

An important part of any modern trauma care system is the establishment, maintenance and continuous development of prehospital Emergency Medical Services (EMS). For trauma patients in particular, rapid transport of the patient to the correct surgical facility is of utmost importance. In Sweden, prehospital care is expected to meet the same fundamental quality requirements as hospital-based care. Currently, prehospital trauma care in Sweden is largely based on the Prehospital Trauma Life Support (PHTLS) concept of operation and Swedish ambulances are staffed by at least one registered nurse (RN) [1].

There are different strategies and concepts regarding how, and how quickly, patients should be assessed and treated in the prehospital setting, and when they should be transported to definitive care. This depends on how the healthcare system in a specific country or region is organized, which involves not only the emergency medical system but also the level of hospital care available at a given time. On a conceptual level there are essentially two extremes, “stay-and-play” or “load-and-go” [2]. In most developed nations, this means that prehospital care could differ depending on patient and accident related circumstances and the availability of different prehospital care resources, from minimal interventions by EMS personnel followed by ground ambulance transport to advanced helicopter emergency medical services (HEMS) taking the patient to a specialized trauma centre [3].

Previous studies looking at prehospital times are numerous and have been performed in several different nations, however, the results are generally quite heterogenous. Some studies have reported a positive effect vis-à-vis survivability when prehospital times are minimized [4, 5]. On the other hand, some studies have found that trauma patients are more likely to survive if the prehospital time was longer and some studies have reported no association at all between prehospital times and mortality [6, 7]. Given the heterogenous results of previous studies, and a relative lack of studies on prehospital times and their effect on patient outcomes in Sweden, the effects of prolonging prehospital times in this setting, remains unclear. Additionally, according to a report from the Swedish National Board of Health and Welfare there is a deficiency with respect to quantitative research in the area of prehospital emergency care [1].

### Aim

The present study aimed to investigate the correlation between prehospital time and mortality among hypotensive trauma patients in Sweden.

## Methods

### Study design, observation period and ethics

The present study was a registry-based retrospective study, with an observation period of 10 years (2013–2022). Prior to initiation, this study was approved by the Swedish Ethical Review Authority which waived the need for informed consent (reference number: 2020–04246).

### Setting

Covering almost 530 000 square kilometres, Sweden is an oblong country located in northern Europe. With a population totaling more than 10.5 million people, nearly 90% of whom live in cities, Sweden is highly urbanized [8].

Data from the Swedish Trauma Registry (SweTrau) was used as the basis for this study. SweTrau is a nationwide registry of trauma patients and at present, of the 49 hospitals in Sweden receiving serious trauma patients, 48 are connected to SweTrau. The registry includes all patients who experienced a traumatic event and were a trauma team was activated, all patients with a New Injury Severity Scale (NISS) score of > 15 even if a trauma team wasn’t activated and patients who are transferred to the hospital within seven days after a traumatic event and had a NISS score > 15. Patients where a trauma team was activated without the patient having experienced a traumatic event or “patients where the only traumatic injury is a chronic subdural hematoma” (SDH) are excluded from the registry. [9] The data entered into SweTrau includes patient characteristics, mechanisms of injury, International Classification of Diseases (ICD) diagnostic codes, ISS and NISS scores, total prehospital time (TPT), response time (RT, time from dispatch to EMS arrival on scene), on-scene time (OST), transport time (TT, time from when the ambulance leaves the scene to arrival at the hospital), other EMS information and information regarding patient outcomes and hospital discharge.

In Sweden, when someone calls the emergency number, a dispatch operator answers, if deemed necessary based on the initial interview with the caller, the dispatch operator may initiate a so called priority-1-alarm [1]. In addition to ambulances staffed by RNs, there are several physician-staffed prehospital emergency care resources available in Sweden. These include nine HEMS bases and physician-staffed rapid-response vehicles. In these cases, the physicians are usually consultant intensive care and anaesthesiology specialists but there are also emergency care physicians working in the prehospital setting in Stockholm and Gothenburg.

### Participants

**Fig. 1:**
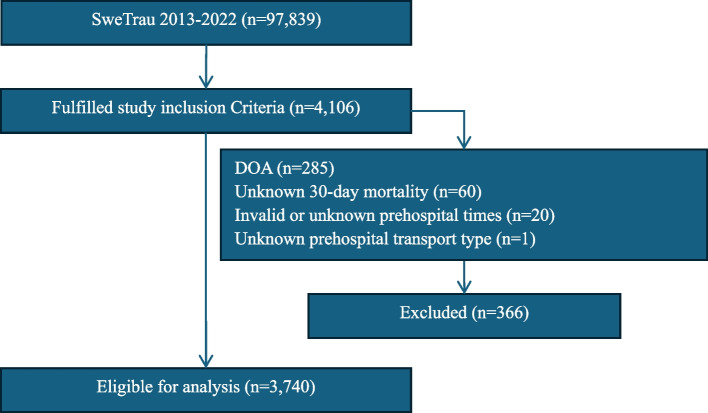
Flow chart of the study inclusion and exclusion process

This study included all adult patients (aged ≥ 15 years) registered in SweTrau between 2013 and 2022 who were hypotensive (SBP ≤ 90 or were categorized as having a RTS SBP category of ≤ 3) at the scene. Patients considered dead-on-arrival (DOA) in the SweTrau database, without vital signs compatible with life, were excluded from the study. Patients with missing, unknown or incorrect outcomes and/or prehospital times were also excluded, as shown in Fig. 1. The study population characteristics are shown in Table 1.

**Table 1:** Baseline characteristics of the study cohort

Patients (n)		3,740
Age, years (median, Q1-Q3)		47 [28–66]
Sex	Female (n,%)	1,151 (30.8)
	Male (n, %)	2,589 (69.2)
Cause of Trauma	Accident (n, %)	2,533 (67.8)
	Self-inflicted (n, %)	532 (14.2)
	Assault (n, %)	580 (15.5)
	Other (n, %)	14 (0.4)
Dominant injury	Blunt force trauma (n, %)	2,929 (78.3)
	Penetrating Trauma (n, %)	792 (21.2)
Mechanism of Injury	Traffic accident (n, %)	1233 (33.1)
	Gun shot wound (n, %)	177 (4.7)
	Stab wound (n, %)	600 (16.0)
	Hit by blunt object (n, %)	226 (6.0)
	Fall, low energy (n, %)	557 (14.9)
	Fall, high energy (n, %)	757 (20.2)
	Explosion (n, %)	6 (0.2)
	Other (n, %)	165 (4.4)
Vital parameters at the scene	SBP, mmHg (median, Q1-Q3)	80 [70–90]
	RR, BPM (median, Q1-Q3)	20 [16–25]
	GCS (median, Q1-Q3)	14 [12–15]
Total prehospital time, min (median, Q1-Q3)		49 [36–66]
Response time, min (median, Q1-Q3)		11 [7–17]
On-scene time, min (median, Q1-Q3)		20 [13–29]
Transport time, min (median, Q1-Q3)		15 [10–24]
NISS (median, Q1-Q3)		17 [5–33]
NISS group	NISS ≤ 8 (n, %)	1110 (30.4)
	NISS 9–15 (n, %)	656 (18.0)
	NISS 16–24 (n, %)	557 (15.3)
	NISS ≥ 25 (n, %)	1329 (36.4)
30-day mortality (n, %)		915 (24.5)

### Variables

All variables used as the basis for this study were gathered from the SweTrau registry.

NISS a scoring system based on the anatomic localization and severity of an injury. Each injury is then assigned an Abbreviated Injury Scale (AIS) score based on their level of severity. The AIS score for the three most severe injuries is squared, and the sum of these three scores equates to the final NISS score. The trauma is considered major when NISS is > 15 [10]. For the basis of the present study, the NISS values registered in SweTrau were used, no additional calculations were made.

RTS is a weighted summation of two physiological vital parameters, systolic blood pressure (SBP) in mmHg and respiratory rate (RR) in breaths per minute as well as neurological status by using Glasgow Coma Scale (GCS) for the latter. In the case of RTS, SBP, RR and GCS is assigned a value between 4 and 0 depending on clinical measurements and examinations [11].

The DOA variable is based on a combination of prehospital cardiac arrest, a prehospital GCS value of 3 and SBP and RR values of 0 according to RTS.

If available in SweTrau, values for these were based on prehospital measurements and examinations. On an individual variable basis, if one or more of these values were missing for a specific patient (*n* = 468) but were obtained in the emergency department (ED) for said patient (*n* = 272), the first value/s obtained in the ED was used instead. Patients with missing values for any of the abovementioned variables, after the addition of ED values, were excluded from the logistic regression analyses (5.2% of patients).

### Study end points

The primary endpoint in this study was 30-day mortality after the initial traumatic event.

### Statistical methods

All statistical analyses in the present study were performed using IBM SPSS Statistics version 29.0.1.0.

Initially, descriptive analyses were performed. Among numerical variables, test of normality was performed using the Kolmogorov–Smirnov goodness-of-fit test, non-normally distributed numerical variables are henceforth presented as medians with interquartile ranges (IQRs), normally distributed numerical variables with means and standard deviation (SD) and categorical variables as frequencies and percentages.

A binary logistic regression analysis was performed to evaluate a potential correlation between the total prehospital time and the study endpoint. Nagelkerke R^2^ was used to evaluate the model, when adjusted for factors other than prehospital believed to influence the outcome; patient age, gender, mechanism of injury, RTS and NISS.

A *p*-value of ≤ 0.05 was considered statistically significant.

For the binary logistic regression analysis performed in the present study, patients with missing data for the variables used to control for confounding mentioned above (7.9 percent of patients, *n* = 295) were excluded from these analyses.

## Results

SweTrau includes a total of 97,839 patients, covering a period of 10 years (2013–2022), of these 3,740 were found to be eligible for analyses, as seen in Fig. 1. While 4,106 patients fulfilled the study inclusion criteria (≥ 15 years of age and hypotensive at the scene), however a total of 366 patients were excluded from the study due to being considered DOA, had an unknown primary outcome (30-day mortality), had unknown or invalid prehospital times or an unknown mode of prehospital transport.

### Main results

**Fig. 2:**
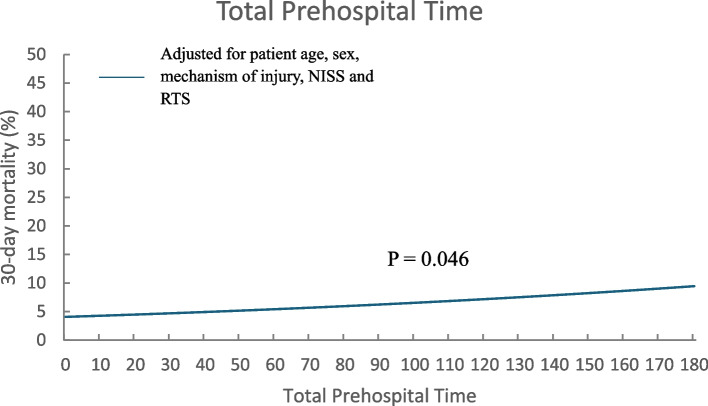
Predicted 30-day mortality according to total prehospital time, adjusted for patient age, sex, mechanism of injury, NISS and RTS, median values used. Adjusted for patient age, sex, mechanism of injury, NISS and RTS

The logistic regression analysis included 3,445 patients and, as shown in Table 2, there was a statistically significant correlation between a longer TPT (*p* = 0.046) and increased odds of 30-day mortality. A higher NISS value and a lower RTS value and an older age also significantly increased the odds of mortality. Figure 2 shows the predicted 30-day mortality with patient age, sex, mechanism of injury, NISS and RTS set to their respective median values in the study population.

**Table 2:** Adjusted logistic regression table

Prehospital time interval	AOR (95% CI)	Significance
Total prehospital time	1.005 (1.000–1.009)	0.046
Patient age	1.049 (1.042–1.057)	< 0.001
NISS	1.040 (1.033–1.047)	< 0.001
RTS	0.478 (0.452–0.506)	< 0.001
Patient sex		
Female	1.280 (0.978–1.675)	0.072
Male	Reference	Reference
Mechanism of injury		
Blunt force trauma	Reference	Reference
Penetrating trauma	1.351 (0.946–1.928)	0.098

The Nagelkerke R^2^ for the logistic regression model was 0.665.

## Discussion

The present study, which aimed to investigate the correlation between prehospital time and survival among hypotensive trauma patients in Sweden, found that a longer total prehospital time was associated with increased mortality among hypotensive trauma patients after adjusting for patient age, sex, mechanism of injury, NISS and RTS. This is in line with some previous studies, though neither of these studies looked specifically at hypotensive patients [12, 13]. A one-minute increase in TPT was found to have an adjusted odds ratio (AOR) for mortality of 1.005 (95% CI 1.000–1.009; *P* = 0.046), which means that a 10-min increase in TPT increases the odds of mortality by 5.1%.

The Nagelkerke R^2^ for the logistic regression analysis in the present study was 0.665, which means that the independent variables included in the model explains close to two thirds of the observed variation in mortality. The model that included all previously mentioned potentially confounding factors, including patient age, NISS, RTS, mechanism of injury and patient sex, in addition to the total prehospital time, was also the model with the highest Nagelkerke R^2^, which means said model was used.

While there are other studies looking specifically at hypotensive trauma patients and subgroup analyses have been performed on hypotensive patients in other studies, the results are quite heterogenous [5, 7, 13]. In the present study, in the adjusted binary regression model, a longer TPT were associated with an increased odds of mortality for undifferentiated trauma patients. Shibahashi et al. looked at a similar population but unlike the present study, they found no associations between prehospital times and mortality [7]. Similarly, in a subgroup analysis of hypotensive patients by Waalwijk et al., they found no associations between mortality and prehospital times [13]. Swaroop et al. did find such an association, though said study looked specifically for hypotensive penetrating trauma patients [5]. Generally, the prehospital times observed in the studies mentioned were quite similar to those in the present study, with, Swaroop et al. possibly considered an exception, with a mean TPT of 27 min [5].

One important distinction is that the abovementioned studies were all performed in different countries, with different guidelines, EMS- and trauma-care-systems. Compared with studies performed in other Scandinavian countries, the results were similar to those observed in the present study, although those studies looked at trauma patients in general, not specifically those who are hypotensive in the prehospital setting [12, 14]. Clarke et al. also found that for hypotensive trauma patients, a longer delay in the ED is associated with an increased probability of mortality, while not a prehospital study, which highlights the importance of getting hypotensive trauma patients to definitive care as soon as the situation allows [15].

Wyen et al. found that several factors impact the prehospital time for trauma patients, including different interventions performed by EMS personnel as well as environmental circumstances, patient characteristics and the type of accident [16]. In said study endotracheal intubation (EI), volume administration and establishing a chest tube all significantly increased OST, as did the patient being an occupant of a car, the patient having an open fracture and darkness. If the patient had suffered penetrating trauma, OST was instead decreased compared to the baseline time of 16 min. Of note, the median OST observed in the present study was 20 min, as seen in Table 1. While the specific underlying cause for a prolonged OST, and as an extension of this the TPT, is outside the direct scope of the present study, this highlights the multifactorial aspects of prehospital times in general and OST in particular.

With the present study being set in Sweden, according to Scandinavian guidelines for management of patients with severe head injuries, an artificial airway should be established in patients with a GCS of 3–8 and where a long TT is expected [17].

Renberg et al. found that, in Sweden, prehospital EI was associated with an increased odds of mortality compared to patients intubated in the emergency department (ED) when adjusted for ISS [18]. In this observational study, they also found that a majority of prehospital intubations were performed by physicians as compared to RNs. The results from said study also suggest that the experience of the airway provider was not the primary driver of mortality among trauma patients intubated prehospitally. Similarly to other studies mentioned, they also found that median OST was significantly longer in cases where prehospital EI was indicated and performed.

While OST may be increased by certain prehospital interventions, Lansom et al. found that trauma patients intubated in the prehospital setting, perhaps unsurprisingly, had a decreased time from arrival at the ED to a computed tomographic (CT) scan being performed when compared to trauma patients who were intubated in the ED [19]. Indeed, Clarke et al. found that time in the ED actually had a greater impact on patient mortality compared to prehospital time [15]. This, and the Nagelkerke R^2^ value of the logistic regression model in the present study, and other studies, show that many factors outside prehospital time that does impact trauma patient mortality, which could help explain some of the inconsistences in results from studies examining the association between prehospital times and patient outcomes [12].

Wyen et al. found that OST has remained largely unchanged over the last two decades [16]. This is quite interesting as there are several studies, including the present study, that shows that a prolonged TPT is associated with an increased odds of mortality for trauma patients. Additionally, studies performed in the hospital setting that shows that the risk of mortality increases the longer it takes for a hypotensive trauma patient to get to definitive care [15].

That total time from injury to definitive care should be as short as possible for trauma patients is hardly a controversial statement [20]. Though the question of what interventions should be performed in the prehospital setting, and therefor what constitutes an optimal on-scene time for trauma patients, remains unanswered [21].

## Limitations

The present study is a registry-based retrospective study, in large registry-based studies there is a potential for type 1 errors. Additionally, in registry-based retrospective studies the risk for selection bias can never be excluded. In the present study, patients considered dead-on-arrival, were excluded, which constitutes an additional risk of selection bias.

To handle missing prehospital data in the calculation of RTS, a next-observation-carried-backwards (NOCB) approach was used, meaning that values obtained in the ED were used instead (for RR and GCS specifically). NOCB has well known deficiencies and is a potential source of bias [22]. However, with the NOCB being used for two (of a total of seven) variables used to control for confounding factors, for 7,3% of patients, the risk of potential bias is considered reasonable, especially considering the comparatively high Nagelkerke R^2^ of 0.665 for the logistic regression model in the present study.

The present study was performed on a Swedish trauma population and within the context of the Swedish prehospital- and trauma-care-system, thus, while the results of this study may be of interest to these instances, the validity of the results outside these contexts haven’t been established. However, with overlapping prehospital guidelines and with the present study showing similar results to studies performed in Norway the results could potentially be applied in a more general, Scandinavian, context, although with some caution and with geographical as well as procedural differences in mind [12, 14, 17].

## Conclusion

In this nationwide study aimed at investigating the correlation between prehospital time and survival among hypotensive trauma patients in Sweden, a longer total prehospital time was found to be associated with increased odds of mortality for undifferentiated hypotensive trauma patients, with the odds of mortality increasing by 5.1% per 10-min increase in total prehospital time.

## Data Availability

The ethical approval does not permit the datasets generated and analysed in the present study to be made publicly available but select data will be made available from the corresponding author at a reasonable request.

## Abbreviations

EMS: Emergency Medical Services
PHTLS: Prehospital Trauma Life Support
RN: Registered Nurse
HEMS: Helicopter Emergency Medical Services
SweTrau: The Swedish Trauma Registry
NISS: New Injury Severity Score
SDH: Subdural Hematoma
ICD: International Classification of Diseases
ISS: Injury Severity Score
TPT: Total Prehospital Time
RT: Response Time
OST: On-Scene Time
TT: Transport Time
GOS: Glasgow Outcome Scale
RTS: Revised Trauma Score
AIS: Abbreviated Injury Scale
SBP: Systolic Blood Pressure
RR: Respiratory Rate
GCS: Glasgow Coma Scale
ED: Emergency Department
IQR: Interquartile Range
SD: Standard Deviation
EI: Endotracheal Intubation
